# Textiloma, migration of retained long gauze from abdominal cavity to intestine

**Published:** 2010

**Authors:** Hojjat Molaei Govarjin, Mohsen Talebianfar, Farinaz Fattahi, Mohammad Esmaeil Akbari

**Affiliations:** a Department of General Surgery, Shohada Teaching Hospital, Shahid Beheshti University Of Medical Sciences, Tehran, Iran; b Cancer Research Center, Shahid Beheshti University Of Medical Sciences, Tehran, Iran; c Professor of Surgery, Shahid Beheshti University Of Medical Sciences, Tehran, Iran

**Keywords:** Surgical Error, Retained Surgical Towel, Gossypiboma, Foreign Body

## Abstract

Retaining of gauzes and surgical sponges in the abdomen is one of the most frequent medical errors usually manifesting as abscess or abdominocutaneus fistulas with no definite symptoms during lifetime. Here, we introduce a 35 year old woman with symptoms and signs of partial bowel obstruction and enterocutaneous fistulas caused by migration of retained gauze from abdominal cavity to terminal ileum, 9 months after cesarean section. This is called “Textiloma”. There are several reports of gossypiboma worldwide but migration of retained gauze into intestine causing an enterocutaneous fistula is rare.

Textiloma (from Latin textile, a woven fabric, plus the suffix oma, meaning swelling or tumor), gossypiboma (from Latin Gossypium, the genus of cotton plants, plus borna, a Kiswahili term meaning place of concealment) and gauzoma (from surgical gauze) are the historical terms referred to pseudo-tumor formation and inflammatory reaction caused by a foreign body or retained non absorbable cotton matrix left behind mistakenly in patient’s body. They usually caused by abdominal and gynaecological surgeries. Despite of being rare, gossypiboma should be considered as differential diagnoses of any mechanical obstruction in patient’s underwent abdominal surgery.^1^–^3^ Manifestations of textiloma are either exudative (abscess formation and granoloma around the surgical sponge or gauze in relation with or without bacterial invasion occurs shortly after surgery) or aseptic (adhesions or encapsulation months/years after surgery of sub acute bowel obstruction). In some sever cases it ends up with bowel perforation, infection and even death. Intra abdominal gauzes can also migrate completely into gastro intestinal lumen (ileum, colon, stomach) or bladder without any opening in the wall. If they are too big, they cannot pass the illeocecal valve and cause partial or complete obstruction.^1^,^3^–^11^

There are several reports of gossypiboma worldwide but migration of retained gauze into intestine causing an enterocutaneous fistula is rare. In this report, we introduce a 35 year old woman with bowel obstruction due to migration of retained gauze into ileum.

## Case Report

**Figure 1:**
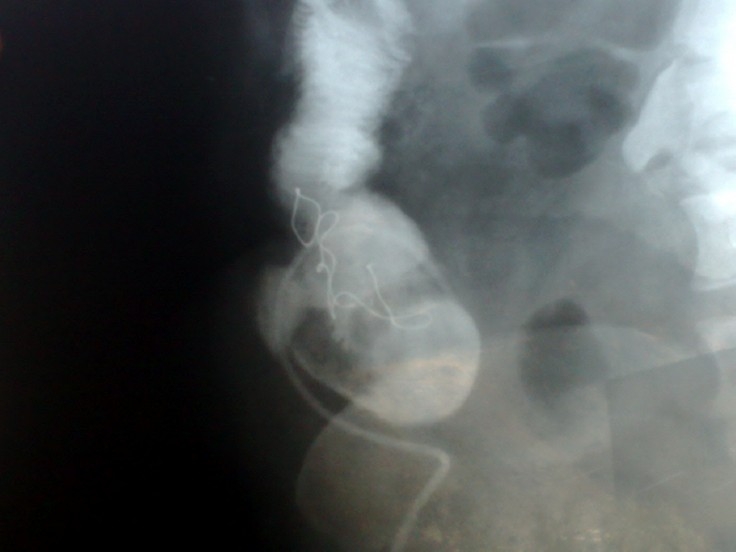
Patient’s fistulography showing retained gauze in intestine

The patient was a 35 year old, G2 L2 A0 female who had discharged from cuteness fistula, frequent abdominal pains 5 months after cesarean section of her second child with Pfannenstiel incision. She was hospitalized with chief complain of anorexia, low grade fever, cutaneus fecal discharges and symptoms of partial bowel obstruction. Patient’s hemodynamic was stable. In her abdominal examination, she had periumblical tenderness but rebound tenderness and mass were not detected. There was a periumblical fistula with fecal discharges. Results of rectal and vaginal exam were normal. She had leukocytosis in CBC diff but other lab test results were normal. She had received complete bowel rest, parenteral nutritional support and antibiotics. Plain x-ray of abdomen showed dilated loops of small bowel with air- fluid levels and opacity in terminal ileum. In fistulography, there were evidences of fistula between small intestine and skin with unusual gas pattern in bowels and retained gauze in terminal ileum (Figure 1). Findings of laparotomy were fistulas between Caecum, terminal ileum and skin with sever intra abdominal adhesions because of retained gauze migration into small intestine. The patient underwent entrolysis, terminal ileotomy and right hemicolectomy with ileocolic anastomoses and removal of fistula tract. Ovaries, ureters and uterus were normal. We repaired fascia and skin with secondary closure. Post operative course was uneventful. Patient was discharged after well toleration of postoperative diets.

## Discussion

Despite all considerations during operation, retained gauzes are still one of the major problems. Textiloma or gossypiboma usually occurs in one out of 100 to 3000 of all surgical investigations and one out of 1000-15000 intra abdominal operations especially with busy surgical fields or emergencies, unplanned changes in procedures and high body mass index.^1^,^12^–^16^

They can cause several complications including intestinal fistula which are important in the prognoses of the disease. Although these complications can be Fatal, they are less reported because of the legal issues.^1^–^3^,^17^

A fistula is an unusual connection between two different surfaces. There are two types of fistula, internal (between digestive tract and other abdominal viscera) and external (includes skin or other epithelial surfaces). About 80% of external fistulas are caused by either medical errors or diseases such as Crohn’s disease and cancers.

Except for FRIENDS (foreign body in fistula duct, radiation, infection, inflammation in ori-gin of fistula, epitheliaziation of fistula duct, neoplasia of fistula and distal obstruction of fistula) most of enterocutaneous fistulas are closed spontaneously. There is usually a 10% recurrence with surgery. In patients with previous history of surgery or palpable mass in site of surgery, granuloma due to foreign body should be considered as a late manifestation.^18^ Plain abdominal radiography, sonography, fistulography, CT scan and MRI are useful for diagnoses.^3^,^5^,^12^–^14^,^19^

In cases that the sponge does not have any radiological marker, it can’t be diagnosed by radiologic screening and may mimic radiographic patterns of hematoma, neoplasm, granulomatous process, abscess formation, cystic masses, calcification and air bubbles as well. The exact modalities of granoloma due to gauze are best seen in contrast enhanced MRI.^20^

There are only a few reports of complete migration of gossypiboma into intestine. Gencosmangulu et al reported abscess formation and severe intraluminal adhesions due to migration of laparotomy towel into midabdomen 2 years after open cholecystectomy and hernia repair in a 74 year old woman presented as small bowel obstruction. They concluded that gossypiboma should be considered as a cause for intestinal mechanical obstruction.^3^

Silva CS et al reported complete migration of retained surgical sponge from abdominal cavity into ileum in a 24- year- old woman 4 months after caesarean section manifested with diffuse colic abdominal pain, nausea, vomiting and constipation. The patient underwent ileotomy with terminating anastomosis. No fistula or open intestinal wall was reported.^4^

Gossypiboma should be removed as soon as diagnosed. Surgery either by laparoscopy or laparotomy is the treatment of choice especially in cases with deeply located foreign body or fistulas. In some asymptomatic cases with complete intraluminal retained gauze, follow up with endoscopy or imaging is recommended.

Yet, prevention is the best treatment. Cooperation of surgical team for exact controlling of all tools and gauzes before ending the operation, intra operative radiologic screening and routine radiography of high risk patients at time of discharge are necessary to prevent further morbidities and legal issues.^3^,^14^,^21^–^23^
